# Modelling of Fatigue Delamination Growth and Prediction of Residual Tensile Strength of Thermoplastic Coupons

**DOI:** 10.3390/ma17020362

**Published:** 2024-01-11

**Authors:** Niki Tsivouraki, Konstantinos Tserpes, Ioannis Sioutis

**Affiliations:** Laboratory of Technology & Strength of Materials, Department of Mechanical Engineering & Aeronautics, University of Patras, 26500 Patras, Greece; niki.tsivouraki@ac.upatras.gr (N.T.); sioutis.i@ac.upatras.gr (I.S.)

**Keywords:** thermoplastic composites, delamination, residual strength, cohesive zone modeling, progressive damage modeling, finite element analysis

## Abstract

Thermoplastic composites are continuously replacing thermosetting composites in lightweight structures. However, the accomplished work on the fatigue behavior of thermoplastics is quite limited. In the present work, we propose a numerical modeling approach for simulating fatigue delamination growth and predicting the residual tensile strength of quasi-isotropic TC 1225 LM PAEK thermoplastic coupons. The approach was supported and validated by tension and fatigue (non-interrupted and interrupted) tests. Fatigue delamination growth was simulated using a mixed-mode fatigue crack growth model, which was based on the cohesive zone modeling method. Quasi-static tension analyses on pristine and fatigued coupons were performed using a progressive damage model. These analyses were implemented using a set of Hashin-type strain-based failure criteria and a damage mechanics-based material property degradation module. Utilizing the fatigue model, we accurately foretold the expansion of delamination concerning the cycle count across all interfaces. The results agree well with C-scan images taken on fatigued coupons during interruptions of fatigue tests. An unequal and unsymmetric delamination growth was predicted due to the quasi-isotropic layup. Moreover, the combined models capture the decrease in the residual tensile strength of the coupons. During the quasi-static tension analysis of the fatigued coupons, we observed that the primary driving failure mechanisms were the rapid spread of existing delamination and the consequential severe matrix cracking.

## 1. Introduction

Composite materials and especially Carbon Fiber Reinforced Plastics (CFRPs) have become the major structural material in aerospace applications [1]. Although in most applications thermoset CFRPs have been used, thermoplastics find an increasing use due to specific characteristics such as increased impact performance, recyclability, and weldability. However, the benefits of thermoplastics in weight reduction and the lowering of recurring costs in aircraft production can be achieved only through the integration of several disciplines and the production of large integral parts. This means that focusing on small parts alone cannot achieve the full benefit of thermoplastic technology. In 2019, the Clean Sky 2 project STUNNING (Next Generation Multifunctional Fuselage Demonstrator) “https://www.clean-aviation.eu/media/results-stories/the-next-generation-multifunctional-fuselage-demonstrator-leveraging-thermoplastics-for-cleaner (accessed on 25 November 2023)”, aiming to build an 8 m long narrow-body airliner fuselage from thermoplastic composites, was launched. After four years of development, a full thermoplastic fuselage part has been built using innovative manufacturing methods and incorporating novel joining techniques such as welding and co-consolidation. Characterization of the mechanical performance of the fuselage part is scheduled for the next phase of the program. In this process, the availability of efficient numerical models capable of simulating fatigue damage and predicting the residual strength of thermoplastics and their joints is of crucial importance, as it could lead to a significant reduction of characterization time and cost.

Delamination, together with matrix cracking, are the primary fatigue failure modes in CFRPs, especially for high cycle fatigue [2]. It is therefore very important to be able to simulate fatigue delamination propagation in CFRPs. Contrary to thermoset CFRPs, there have been reported only a few experimental and numerical works on the delamination of thermoplastics; most of them have considered quasi-static loads. In the first effort, O’Brien [3] has conducted delamination tension fatigue tests on AS4/PEEK composite laminates. Uematsu et al. [4] have studied experimentally the behavior of delamination crack propagation of a carbon-fiber-reinforced thermoplastic polymer at high temperatures. Lachaud et al. [5] have conducted experimental and numerical studies of delamination caused by local buckling on thermoset and thermoplastic carbon-fiber composites. Zhou et al. [6] have described the mode I delamination behavior of a unidirectional carbon-fiber/polyphenyleneetherketone (PEK-C) composite experimentally. Kenny et al. [7] have studied the elastoplastic behavior of thermoplastic matrix composite laminates under cyclic loading. The proposed mathematical model predicted the visco–elastic–plastic response of the material at high stresses and its influence on fatigue damage. Jen et al. [8,9] examined experimental and theoretical tensile and fatigue behavior of thermoplastic composite laminates using an extended Tsai-Hill failure criterion, which satisfying comparison came from. Xiao et al. [10] considered the correlation between fatigue life and thermal degradation of fatigue strength. Dube et al. [11] have performed an experimental investigation characterizing the fatigue failure mechanisms of resistance-welded thermoplastic composites’ skin/stringer joints. Delamination has been found to be a major failure mechanism of the joints. Ruzek et al. [12] have conducted an experimental investigation of the effects of the loading frequency on the temperature change, fatigue behavior, and failure mechanisms of carbon-fiber-fabric-reinforced polyphenylenesulphide (PPS) laminates, the thicknesses of which were varied using ply drops. 

The numerical works are much fewer than the experimental ones and they have mainly considered quasi-static and impact loads. Rajoli et al. [13] have proposed a numerical fatigue model by considering the effect of static damage growth during cyclic loading. Keiichi et al. [14] have investigated the stress–strain response and the damage initiation/propagation mechanisms of T700G/LM-PAEK material, in an open-hole configuration, experimentally and numerically. Liu et al. [15] have developed 3D FE models using the LS-DYNA program to study the effects of impact energy, ply angle, and interfacial strength on the low-velocity impact performance of thermoplastic laminates. Sun et al. [16] have made comparisons between experimental and numerical studies of low-velocity impact damage for thermoplastic (IM7/PEEK) composites. Leciñana et al. [17] have studied the Mode I interlaminar fatigue behavior of thermoplastic composites considering R-curve effects. Three LS-DYNA FE models (standard, continuum damage mechanics (CDM), and discrete) were developed, all using cohesive interface elements for delamination. Recently, Sioutis and Tserpes [18] have developed a fatigue interfacial crack growth model based on the cohesive zone modeling method, which has been proven capable of efficiently simulating the fatigue interfacial fracture of co-consolidated thermoplastic joints. Provided the co-consolidated interfaces have the same mechanical performance as the ply interfaces, the model of Sioutis et al. [18] could be used for simulating fatigue delamination of thermoplastic coupons.

Investigations at the mesoscale and microscale hold equal significance to macroscopic studies because they offer valuable internal perspectives into mechanics. Cao et al. [19] carried out a mesoscale numerical simulation and experimental verification on the bearing failure of single-lap and double-lap thin-ply laminated composite bolted joints. Song et al. [20] proposed a prediction model for plastic deformation of thermoplastic fiber-reinforced composites.

The objective of the present paper is twofold: to examine whether the model of Liu et al. [15] could be used for simulating fatigue delamination propagation and to combine the fatigue model with quasi-static progressive damage models to develop a tool capable of predicting the residual tensile strength of thermoplastic coupons, which when upscaled may be used in the characterization process and the damage tolerant design of thermoplastic structures.

## 2. Experimental Methods

The composite coupons were made from thermoplastic TC 1225 LM PAEK prepregcarbon plies (Unidirectional–UD) with a fiber volume fraction of 66% (Netherlands). The coupons were approximately 250 mm long, 25 mm wide and 2.24 mm thick. Tabs from the same material with dimensions of 50 mm × 25 mm × 2.24 mm were bonded to the coupons. The 0.14 mm-thick plies were stacked in a quasi-isotropic stacking sequence following a layup of [−45/0/45/90]_2s_ [21].

Tension tests were conducted according to the ASTM D3039 standard [22] and fatigue tests according to the ASTM D3479 standard [23]. All tests were conducted using an MTS Universal Testing machine with a load cell of 100 kN and a data acquisition program. Tension tests were conducted in displacement control at a 0.5 mm/min loading rate. Fatigue tests were conducted in force control using a frequency of 5 Hz, a stress ratio of 0.1 and a maximum stress equal to 60% of the static strength.

The following test series were conducted:

1. Five coupons were tested in tension to characterize the reference tensile behavior of the thermoplastic material.
2. Five coupons were tested in fatigue to characterize the reference tension–tension fatigue behavior of the thermoplastic material.
3. Thirteen coupons were tested in fatigue to monitor the progressive fatigue damage through Ultrasonic C-Scan images.
4. Six specimens were subjected to fatigue up to cycles equal to 0.1, 0.2, 0.3, 0.4, 0.5 and 0.6 of the average fatigue life of the coupons that have undergone interrupted fatigue testing.
5. The six fatigued coupons were tested in tension to measure the residual tensile stiffness and strength of the coupons.

**Figure 1:**
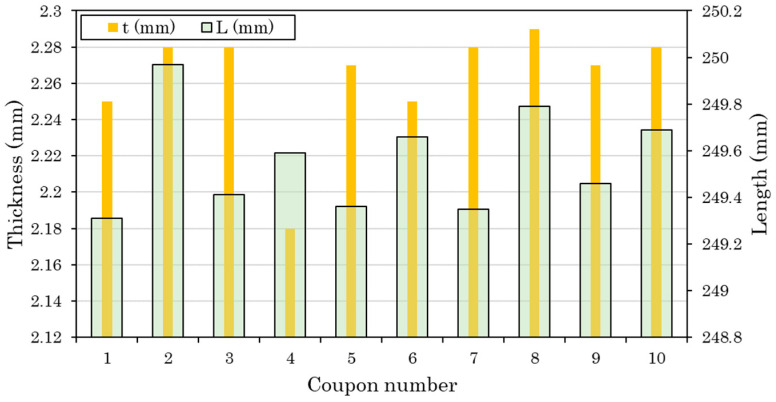
Deviation of identical nominal dimensions (length, thickness) of coupons—indicatively for 10 coupons (tensile and fatigue tests).

In Figure 1 is displayed the deviation of identical nominal dimensions (length, thickness) of 10 coupons indicatively.

## 3. Numerical Procedure

The adopted modeling approach is based on the work of Tserpes et al. [24]. It combines a quasi-static progressive damage model (PDM) [25,26], the cohesive zone modeling (CZM) method, and a fatigue interfacial crack growth model [18]. The PDM is used to simulate intralaminar damage and the CZM method together with the fatigue crack growth model to simulate fatigue delamination. The execution of the models follows the experimental procedure described in Section 2. Initially, the PDM is used to simulate the tensile behavior of the thermoplastic material. Then, the fatigue crack growth model is used to simulate fatigue delamination. Finally, the PDM is used to predict the residual tensile strength of the thermoplastic coupons at various fatigue delamination states. At all stages, the numerical results are compared with the experimental ones for the models’ validation.

### 3.1. Quasi-Static Progressive Damage Model

To simulate the tensile behavior of the thermoplastic coupons, a progressive damage model (PDM) was used. The PDM was realized through the material model MAT-162 [27] of the LS-Dyna, which comprises a set of Hashin-type strain-based failure criteria [28] along with a damage mechanics-based material property degradation module proposed by Matzenmiller et al. [29]. The Hashin-type strain-based failure criteria considered are listed below.

Tension-Shear fiber mode 1

(1) $$f_{1}-r_{1}^{2}={\left(\frac{E_{11}\left\langle \varepsilon_{11}\right\rangle }{S_{1T}}\right)}^{2}+\left(\frac{G_{12}^{2}\varepsilon_{12}^{2}+G_{31}^{2}\varepsilon_{31}^{2}}{S_{FS}^{2}}\right)-r_{1}^{2}=0,$$

Compression fiber mode 2

(2) $$f_{2}-r_{2}^{2}={\left(\frac{E_{11}\left\langle \varepsilon_{11}^{\prime }\right\rangle }{S_{1C}}\right)}^{2}-r_{2}^{2}=0,\varepsilon_{11}^{\prime }=-\varepsilon_{11}-\frac{\left\langle -E_{33}\varepsilon_{33}-E_{22}\varepsilon_{22}\right\rangle }{2E_{11}},$$

Crush mode 3

(3) $$f_{3}-r_{3}^{2}={\left(\frac{E_{33}\left\langle -\varepsilon_{33}\right\rangle }{S_{FC}}\right)}^{2}-r_{3}^{2}=0,$$

Transverse compressive matrix mode 4

(4) $$f_{4}-r_{4}^{2}={\left(\frac{E_{22}\left\langle -\varepsilon_{22}\right\rangle }{S_{2C}}\right)}^{2}-r_{4}^{2}=0,$$

Perpendicular matrix mode 5

(5) $$f_{5}-r_{5}^{2}={\left(\frac{E_{22}\left\langle \varepsilon_{22}\right\rangle }{S_{bT}}\right)}^{2}+{\left(\frac{G_{23}\varepsilon_{23}}{S_{230}+S_{SRB}}\right)}^{2}+{\left(\frac{G_{12}\varepsilon_{12}}{S_{120}+S_{SRB}}\right)}^{2}-r_{5}^{2}=0,$$

Parallel matrix (delamination) mode 6

(6) $$f_{6}-r_{6}^{2}=S_{delm}^{2}\left\{{\left(\frac{E_{33}\left\langle \varepsilon_{33}\right\rangle }{S_{3T}}\right)}^{2}+{\left(\frac{G_{23}\varepsilon_{23}}{S_{230}+S_{SRC}}\right)}^{2}+{\left(\frac{G_{31}\varepsilon_{31}}{S_{310}+S_{SRC}}\right)}^{2}\right\}-r_{6}^{2}=0$$

where 〈 〉 are the Macaulay brackets; *S*_1*T*_ and *S*_1*C*_ are the tensile and compressive strengths in the fiber direction; *S_FS_* and *S_FC_* are the layer strengths associated with the fiber shear and crush failure, respectively; *S*_2*T*_ and *S*_3*T*_ are the transverse tensile strengths of the corresponding tensile modes (*ε*_22_ > 0 or *ε*_33_ > 0); and *S*_120_, *S*_230_, and *S*_310_ are the quasi-static shear strength values. Note that a scale factor S*_delm_* is introduced to provide a better correlation of the delamination area with the experiments. The scale factor *S_delm_* can be determined by fitting the analytical prediction to the experimental data for the delamination area.

Under compressive transverse strain, *ε*_22_ < 0 or *ε*_33_ < 0, the damaged surface is considered to be “closed”, and the shear strengths are assumed to depend on the compressive normal strains based on the Mohr-Coulomb theory

(7) $$S_{SRB}=E_{22}\tan\left(\varphi \right)\left\langle -\varepsilon_{22}\right\rangle$$

(8) $$S_{SRC}=E_{33}\tan\left(\varphi \right)\left\langle -\varepsilon_{33}\right\rangle$$

where *φ* is a material constant as tan(*φ*) is similar to the coefficient of friction. The damage thresholds *r_j_*, *j* = 1, …, 6, have the initial values equal to 1 before the damage is initiated and are updated due to damage accumulation of the associated damage modes.

A set of damage variables ${\bar{\omega }}_{i}$ with *_i_* = 1, …, 6; are introduced to correlate the onset and growth of damage to stiffness losses in the material. The compliance matrix $\left[S\right]$ is related to the damage variables as

(9) $$\left[S\right]=\left[\begin{array}{cc} \begin{array}{ccc} \frac{1}{\left(1-{\bar{\omega }}_{1}\right)E_{11}} & -\frac{v_{21}}{E_{22}} & -\frac{v_{31}}{E_{33}}\\ -\frac{v_{12}}{E_{11}} & \frac{1}{\left(1-{\bar{\omega }}_{2}\right)E_{22}} & -\frac{v_{32}}{E_{33}}\\ -\frac{v_{13}}{E_{11}} & -\frac{v_{23}}{E_{22}} & \frac{1}{\left(1-{\bar{\omega }}_{3}\right)E_{33}} \end{array} & 0\\ 0 & \begin{array}{ccc} \frac{1}{\left(1-{\bar{\omega }}_{4}\right)G_{12}} & 0 & 0\\ 0 & \frac{1}{\left(1-{\bar{\omega }}_{5}\right)G_{23}} & 0\\ 0 & 0 & \frac{1}{\left(1-{\bar{\omega }}_{6}\right)G_{31}} \end{array} \end{array}\right]$$

The stiffness matrix [*C*] is obtained by inverting the compliance matrix, i.e., $\left[C\right]={\left[S\right]}^{-1}$. As suggested in [29], the growth rate of damage variables, ${\bar{\omega }}_{i}$, is governed by the damage rule of the form:

(10) $${\dot{\bar{\omega }}}_{i}=max\left\{{\dot{\varphi }}_{j}q_{ij}\right\},$$

where the scalar damage function ${\dot{\varphi }}_{j}$ controls the amount of growth and the vector-valued matrix $q_{ij}$ (*i* = 1, …, 6, *j* = 1, …, 6) provides the coupling between the individual damage variables (*i*) and the various damage modes (*j*). The damage criteria of Equations (1) to (6) provide the damage surfaces in strain space for the unidirectional and fabric lamina models, respectively. Damage growth, ${\dot{\varphi }}_{j}>0$, will occur when the strain path crosses the updated damage surface $f_{j}-r_{j}^{2}=0$ and the strain increment has a non-zero component in the direction of the normal to the damage surface, i.e., $\sum_{i}\frac{\partial f_{j}}{\partial \varepsilon_{i}}{\dot{\varepsilon }}_{i}>0$. Combined with damage growth functions $\gamma_{j}\left(\varepsilon_{i},{\bar{\omega }}_{i}\right)$, ${\dot{\varphi }}_{j}$ is assumed to have the form:

(11) $${\dot{\varphi }}_{j}=\sum_{i}\gamma_{j}\frac{\partial f_{j}}{\partial \varepsilon_{i}}{\dot{\varepsilon }}_{i}$$

choosing

(12) $$\gamma_{j}=\frac{1}{2}\left(1-\varphi_{j}\right)f_{j}^{\frac{m_{j}}{2}-1}$$

and noting that

(13) $$\sum_{i}\frac{\partial f_{j}}{\partial \varepsilon_{i}}{\dot{\varepsilon }}_{i}={\dot{f}}_{j},$$

for the quadratic functions given by Equations (1) to (6) lead to:

(14) $${\dot{\varphi }}_{j}=\frac{1}{2}\left(1-\varphi_{j}\right)f_{j}^{\frac{m_{j}}{2}-1}{\dot{f}}_{j}$$

Equation (14) is the damage coupling matrix, and Equation (15) and Table 1 explain how it is associated with the modulus reduction for the unidirectional lamina model.

(15) $$q_{ij}^{U}=\left[\begin{array}{cc} \begin{array}{ccc} 1 & 1 & 1\\ 0 & 0 & 1\\ 0 & 0 & 1 \end{array} & \begin{array}{ccc} 0 & 0 & 0\\ 1 & 1 & 0\\ 0 & 0 & 1 \end{array}\\ \begin{array}{ccc} 1 & 1 & 1\\ 0 & 0 & 1\\ 1 & 1 & 1 \end{array} & \begin{array}{ccc} 1 & 1 & 0\\ 1 & 1 & 1\\ 0 & 0 & 1 \end{array} \end{array}\right]\;i=1,\ldots ,6;j=1,\ldots ,6$$

### 3.2. Fatigue Crack Growth Model

It should provide a concise and precise description of the experimental results, their interpretation, as well as the experimental conclusions that can be drawn.

To simulate fatigue delamination growth, a recently developed fatigue crack growth model for co-consolidated thermoplastics [18] was used. The model relies on a modified Paris’ law, which is used for the calculation of fatigue crack growth rate da/dN in form of

(16) $$\frac{da}{dN}=c{\left(G_{max}\right)}^{m}$$

where *c* and *m* are modified Paris’ law parameters at each simulation’s state and $G_{max}$ is the maximum energy release rate of the affected element at the given state. The instantaneous energy release rate Gi for each cohesive element is constantly equal to the maximum energy release rate $G_{max}$ of the real loading spectrum, due to the use of the load envelope technique.

The intermediate mixed-mode *c* and *m* parameters were derived using the prediction model of Russel and Street [30], which requires as input only pure mode I and mode II fatigue data.

The numerical fatigue degradation has been implemented via the method of cumulative static and fatigue element damage parameter given using

(17) $$d_{tot}=d_{s}+d_{f},$$

where $d_{tot}$ is the total damage variable (0: undamaged, 1: failed), while $d_{s}$ and $d_{f}$ are the static and fatigue contributing damage variables, respectively. The stress state *σ* for each element in the fatigue activated zone has been degraded using

(18) $$\sigma =\left(1-d_{tot}\right)\sigma_{max}$$

where $\sigma_{max}$ is the maximum traction of the affected element.

In this study, the modified Paris’ law was used accounting only for $G_{max}$, which has also been used in works of other authors [31,32,33]. The fatigue crack growth model has been fully validated upon the same experimental parameters (stress ratio, max. load percentage) for the same interfaces in [18].

**Figure 2:**
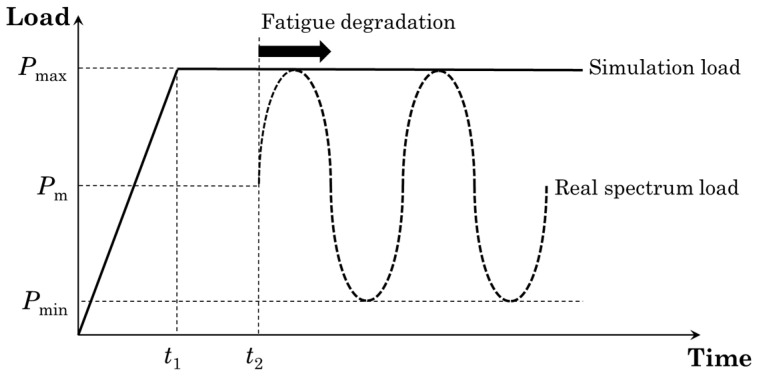
The load envelope for the modeling of fatigue loading.

The fatigue loading was based on the loading envelope technique [34], where the actual sinusoidal spectrum was approached by a constant force which corresponds to the maximum fatigue loading value as can be seen in Figure 2. 

**Figure 3:**
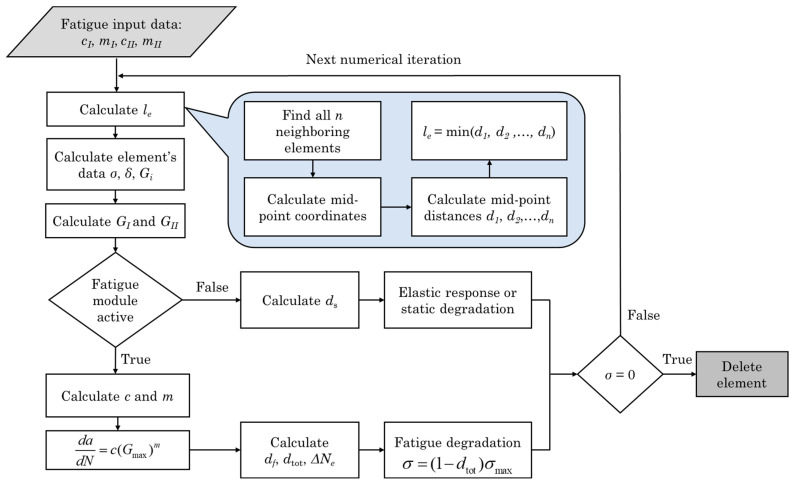
Flowchart of the fatigue crack growth model.

During every numerical iteration, the rate of fatigue crack growth was calculated using Equation (16), which operates in a singular direction. This results in establishing an equivalent rate of damage development uniformly across all axes, disregarding the orthotropic nature of thermoplastics and the intricate nature of the three-dimensional interfaces in the model. To resolve this limitation, a specific length measurement was allocated to individual elements, guiding the progression of damage by considering the input from the cohesive integration points. The significance of this measurement becomes most apparent in scenarios involving substantial diversity in the aspect ratios of elements and irregularities within the interfaces. The calculation of the fatigue damage rate $d_{f}$ requires the values of the characteristic elements’ length $l_{e}$, which is described in detail in [18]. $l_{e}$ was constantly computed (for each iteration) through an internal loop as the minimum distance from the mid-point and non-failed to the neighbor elements’ mid-point. The calculation of the element’s data and G_I_ and G_II_ followed. A double check of activation of fatigue module was subsequent. If G_I_ or G_II_ was over the G_thres_ value, then the fatigue module was activated; otherwise, static degradation was activated. The G_thres_ was based on the available bibliography. The stress *σ* of the cohesive element refers to the mixed mode stress, which accrues from the subcomponents of both the normal and tangential stress of the element, according to LS-Dyna’s MAT_138 formulation [35]. Thus, the cohesive elements subjected to fatigue damage were fully governed by the mixed-mode stress (*σ*) for their response, degradation, and failure. The iterative numerical procedure for the fatigue crack growth model is graphically presented in Figure 3.

**Figure 4:**
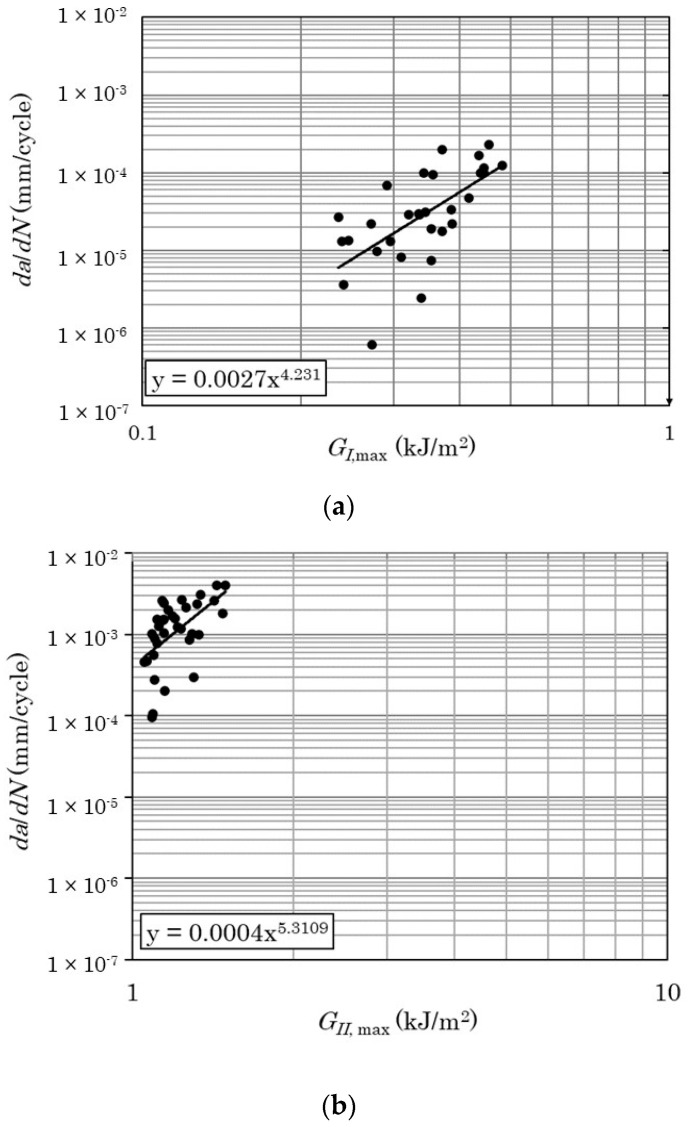
The modified Paris’ law for (**a**) the pure mode I and (**b**) the pure mode II fatigue load cases.

The pure mode I and mode II modified Paris’ laws that have been used in the present application are:

(19) $$({\frac{d\alpha }{dN})}_{I}=0.0027G_{I,max}^{4.23}$$

(20) $$({\frac{d\alpha }{dN})}_{II}=0.00040G_{II,max}^{5.31}$$

where ${\left(\frac{da}{dN}\right)}_{I}$, ${\left(\frac{da}{dN}\right)}_{II}$ and $G_{I,max\;}$, $G_{II,max\;}$ are the fatigue crack growth rates and the maximum energy release rates per cycle for mode I and mode II, respectively. Figure 4 depicts the experimental $\frac{da}{dN_{I}}-G_{max}\;$ from which the modified Paris laws were derived. More details on the derivation of Equations (19) and (20) and the respective graphs in Figure 4 can be found in [18].

### 3.3. FE Models

**Figure 5:**
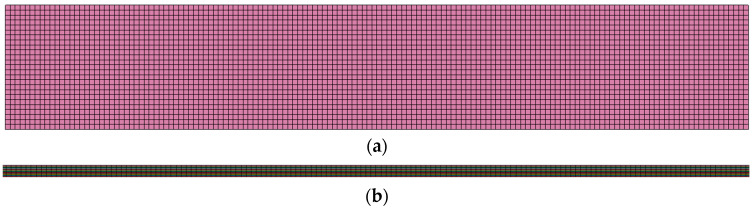
Mesh density of quasi-static tension model (**a**) front view and (**b**) side view.

**Figure 6:**
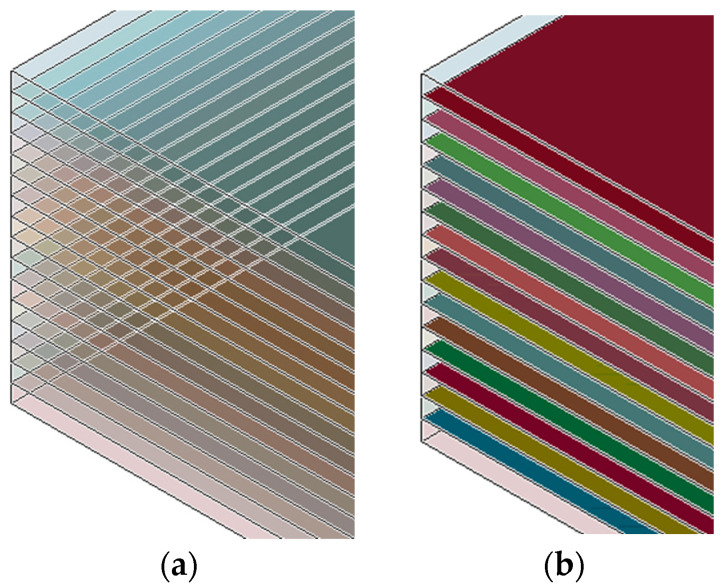
Schematics of (**a**) the orthotropic plies (tension models), (**b**) the cohesive layers (fatigue model).

The specimens modeled had the same dimensions as the coupons tested (see Section 2). 8-noded solid elements were at all FE models. The global damping for the models was specified at 800 for the entire analysis, while a Rayleigh damping coefficient of 0.1 was applied to achieve a higher structural rigidity. Furthermore, to eliminate non-physical hourglass instabilities, a Flanagan–Belytschko integration stiffness formulation was selected with a coefficient of 0.1. The mesh density of the coupon is displayed in Figure 5 in front and side view (element edge length: 1 mm for length and width; 0.14 mm for thickness). The mechanical properties of the thermoplastic layer assigned to the elements are listed in Table 2, which is based on the manufacturer’s datasheet, mechanical experiments, and bibliography. The uncertainty of the material’s properties is due to manufacturing and cutting process. The manufacturer provides nominal values of properties, which needed to be confirmed via mechanical tests, as via the available bibliography. The ply interfaces of the fatigue and residual strength FE models were modeled using intermediate cohesive layers, as Figure 6 is displayed. The cohesive elements were modeled using the UMAT 43 material model [27] designed for three-dimensional cohesive elements, and implemented as zero-thickness elements. The quasi-static tensile load was modeled by fixing the nodes at one end and applying an incremental displacement at the other end. The fatigue load was modeled by increasing the applied displacement until the maximum load was reached. A predetermined time interval was chosen for both fatigue and quasi-static models to reduce computational time without compromising the required level of accuracy.

**Figure 7:**
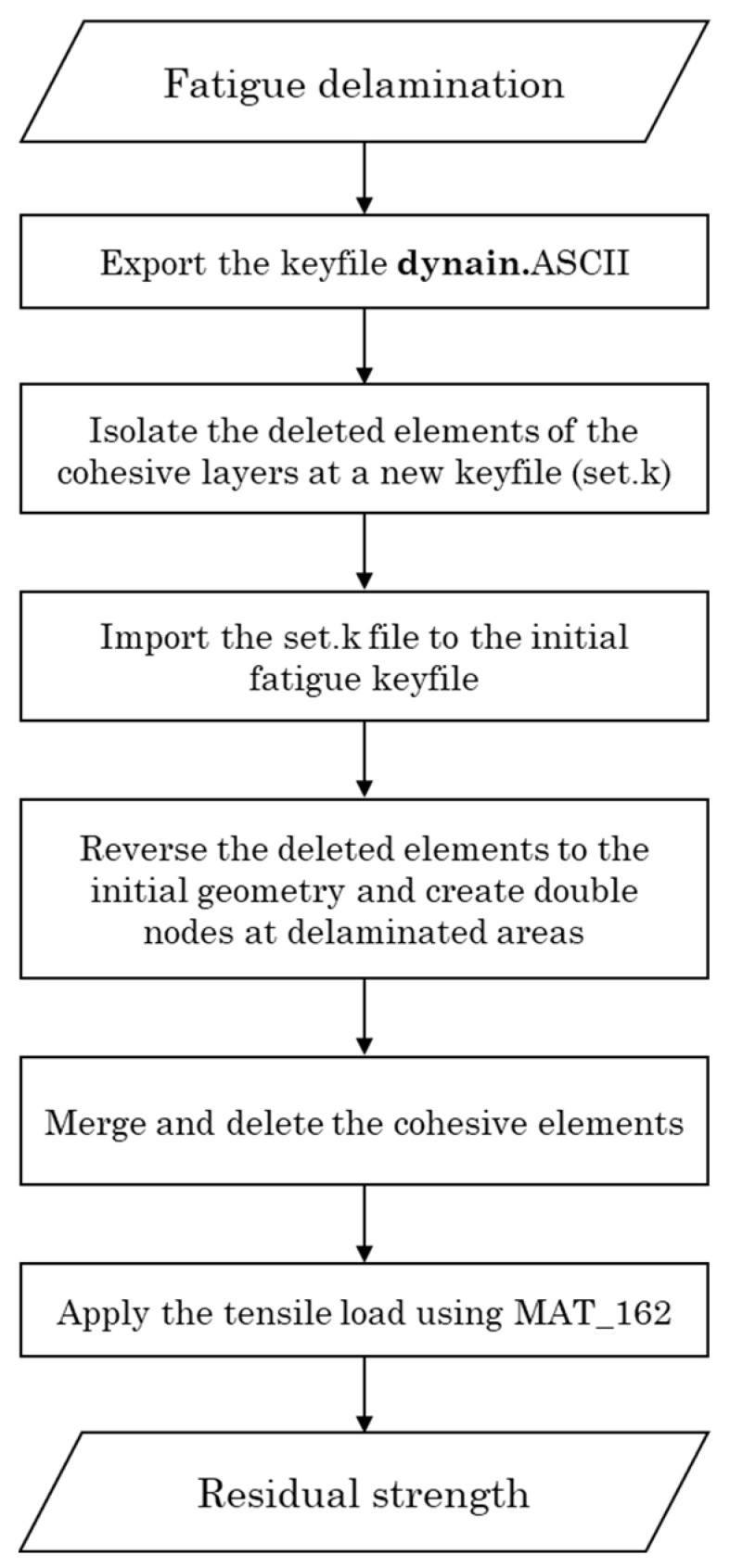
Flowchart of the numerical process of the residual strength model.

To predict the residual tensile strength of the thermoplastic coupons, the PDM and the fatigue model are combined through means of the numerical procedure described in the flowchart of Figure 7. For each fatigue state of interest, a dynain.ASCII keyfile was extracted, encompassing the geometry and the deleted cohesive elements from the fatigue model which delineate the delamination area of the state. Subsequently, the isolated deleted cohesive elements were imported into the initial fatigue model. Following this, the restoration of deleted elements to their original geometry was selected, resulting in the creation of double nodes at locations where delamination had occurred. The cohesive elements were then merged and removed, and tensile loading using MAT_162 (with properties identical to those in the model validation) was applied to extract the residual strength. 

The failure mode of matrix cracking presents a challenge in precise modeling, as it does not conform neatly to the established fatigue model or pre-existing damage state within the residual strength prediction model. The information gleaned from experimental photos and C-Scan data, while valuable, remains insufficient to offer a clear and objective understanding of the evolution of matrix cracking. Consequently, MAT_162 has been chosen as the material model for both quasi-static models. This selection was based on its capability to delineate the evolution of matrix cracking and delamination at each layer, thereby providing a more comprehensive insight into the intricate mechanisms underlying these phenomena.

## 4. Comparison of Numerical and Experimental Results

### 4.1. Tensile Behavior

**Figure 8:**
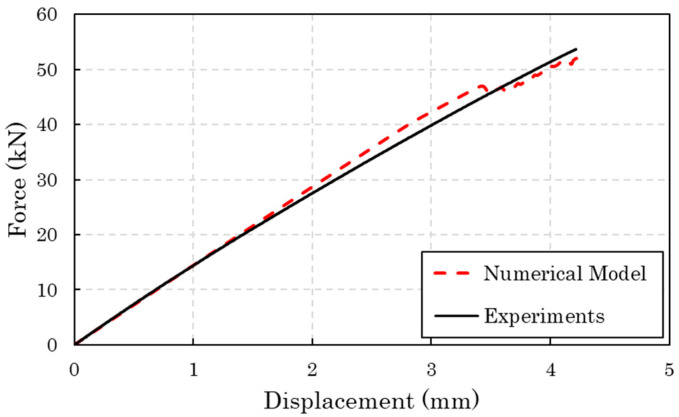
Experimental (5 coupons examined) and numerical tensile force-displacement curves of the thermoplastic coupons.

Figure 8 plots the experimental and numerical tensile force-displacement curves of the thermoplastic coupons. The observed scatter of the experimental curves is acceptable. The average tensile longitudinal strength is 917 MPa and the average longitudinal modulus is 59.75 GPa. Regarding the numerical results, these represent very well the initial stiffness of the coupons, the maximum applied displacement, and the tensile strength. However, they do not capture accurately the decrease in the slope of the curves that takes place after the applied displacement of 1 mm, while the curvature of the experimental curve is due to the visco-elastic behavior of the thermoplastic matrix and cannot be modelled precisely, while knowledge about this behavior is not available from the manufacturer.

**Figure 9:**
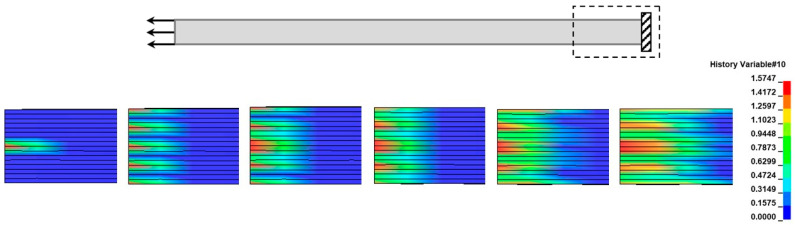
Matrix cracking propagation due to quasi-static tension at different time stages (Examined damage mode: perpendicular matrix mode). At the top of the figure, the monitoring area of the coupon is shown (the black box shows the examined region and the arrows the boundary conditions).

**Figure 10:**
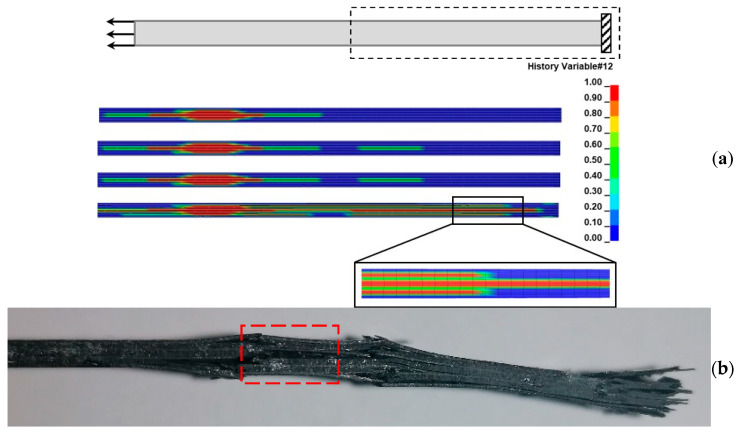
(**a**) Delamination propagation due to quasi-static tension (numerically) at different time stages (the black box shows the examined region and the arrows the boundary conditions (**b**) Delamination in the middle interface (red box) due to quasi-static tension (experimentally).

The main damage modes that drove the tensile failure of the coupons are matrix cracking and delamination. Matrix cracking initiated at 25 kN at the supported end of the coupon, as can be seen in Figure 9. Specifically, it started at the middle 90° layers and propagated to the other 90° and ±45° layers. On the other hand, delamination initiated at 46 kN to a large extent and caused a sudden load drop in the numerical curve, which does not appear in the experimental curves. Delamination started in the middle area of the coupon (Figure 10) at the middle 90°/90° interface and propagated at the 90°/±45° interfaces towards the supported end of the coupon.

### 4.2. Fatigue Life

**Figure 11:**
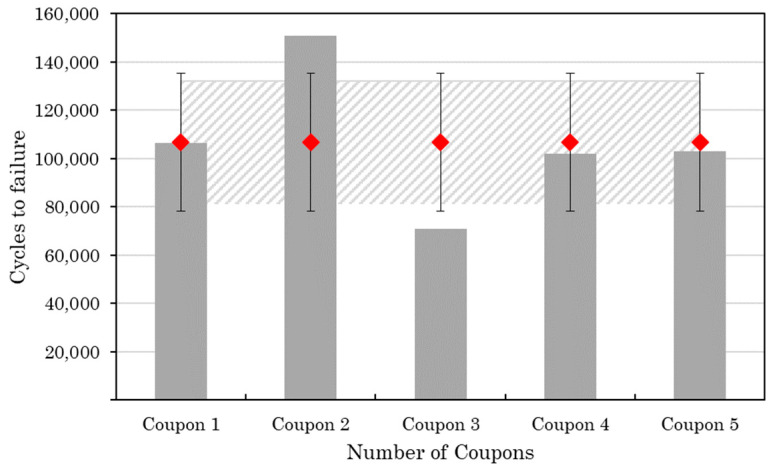
Fatigue lives of the coupons of the non-interrupted tests (red points are the mean value).

**Figure 12:**
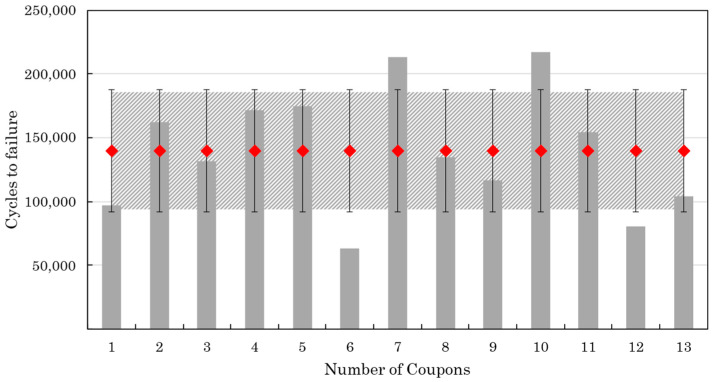
Fatigue lives of the coupons of the interrupted test (red points are the mean value).

Figure 11 summarizes the results from the non-interrupted fatigue tests. The purpose of this test series was to measure the average fatigue life, based on which the settings (number of intervals and number of cycles between intervals) of the interrupted fatigue tests will be determined. The average fatigue life is 106,612 cycles, and the standard deviation is 25,514 cycles. The relatively high standard deviation is attributed to the complexity of the fatigue failure mechanisms and the manufacturing defects which were present in the coupons. Figure 12 presents the fatigue lives of the coupons that have undergone interrupted fatigue testing. In this case, the average fatigue life is 140,037 cycles, and the standard deviation is 46,640 cycles. The increase in the standard deviation compared to the non-interrupted tests is probably due to processing errors that were introduced during the several stops and restarts of the testing process.

### 4.3. Fatigue Delamination 

**Figure 13:**
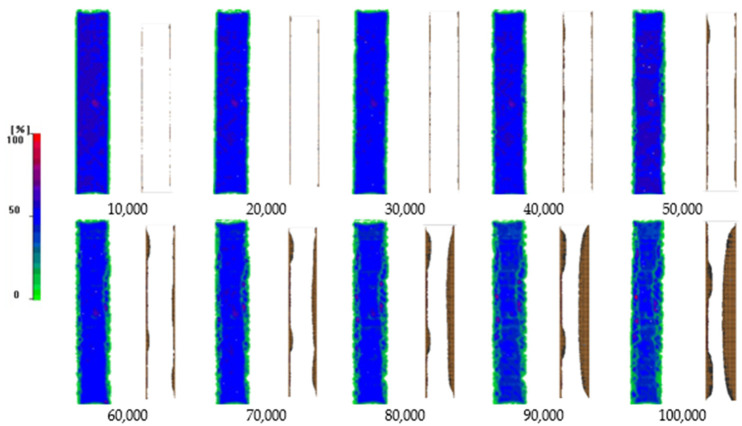
Comparison between the experimental fatigue delamination growth as detected via C-scanning (images with blue and green color–C-Scan color map at left of the image) and numerically (images with white and brown color) predicted (−45/90 interface).

The use of cohesive elements at each interface enables the simulation of delamination propagation as a function of the number of cycles in the entire coupon. For validation purposes, the predicted delamination propagation at the −45°/90° interface is compared with C-scan images taken during interruptions of the fatigue test in Figure 13. As revealed, the model captures accurately both the initiation of delamination at the coupon’s edges at 10,000 cycles and the propagation rate of delamination towards the middle of the coupon. The delamination initiation and propagation in the free edges of composite laminates under fatigue is confirmed also in other studies, such as Bogenfeld et al. [36], in which neighboring 45° interfaces plies are more sensitive and have a tendency to start the examined damage modes.

**Figure 14:**
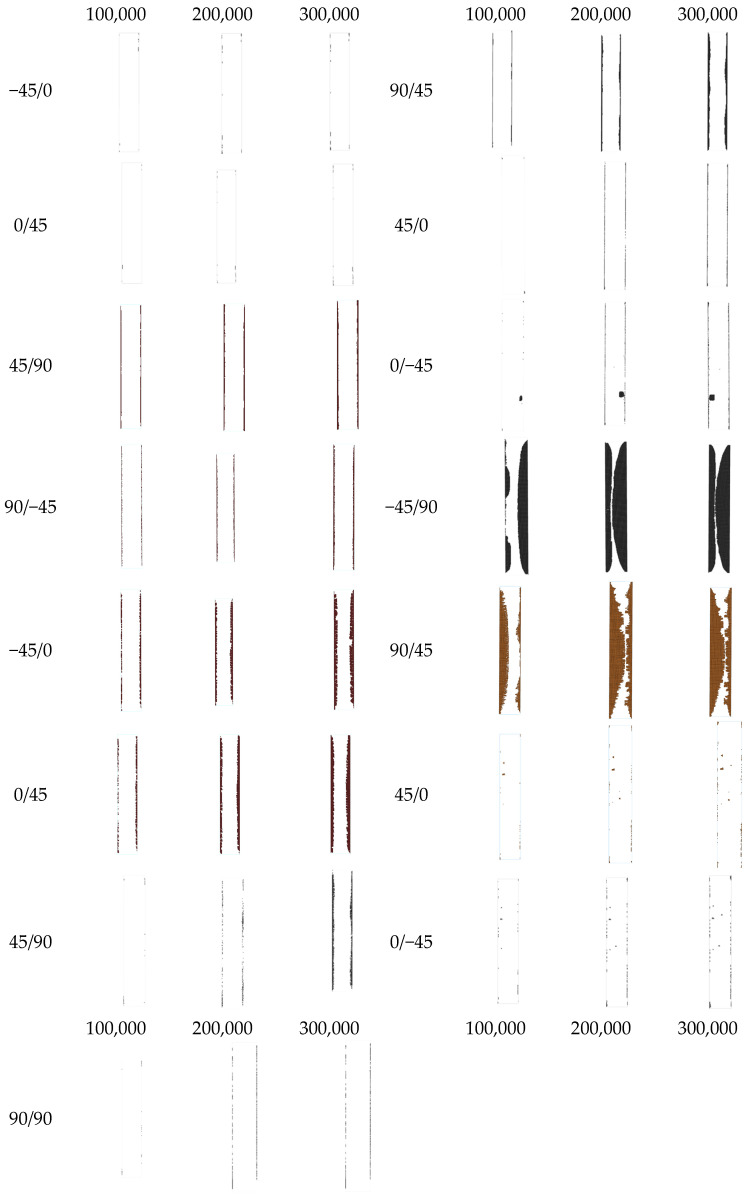
Predicted fatigue delamination growth at different ply interfaces.

**Figure 15:**
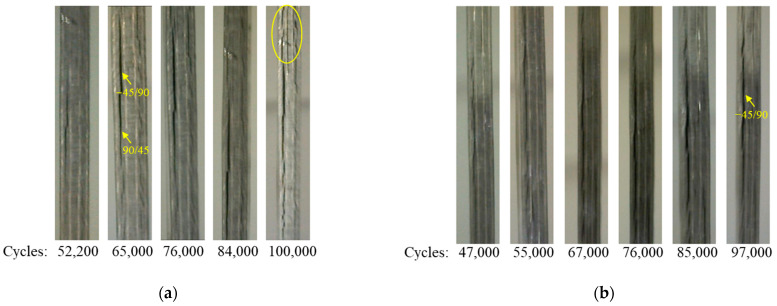
Side-view images at different fatigue stages of (**a**) coupon 13 and (**b**) coupon 10 showing delamination and matrix cracking (yellow oval shape).

Figure 14 depicts the delamination propagation at all interfaces of the coupon for every 100,000 cycles. The quasi-isotropic layup of the coupons has caused an unsymmetric and unequal delamination propagation between the interfaces. Delamination is mainly propagating to the −45°/90° and the 90°/45° interfaces at one side of the coupon and at the interfaces of the 90° layers, while it is almost absent from the interfaces of the 0° layers. The different delamination propagation between the symmetric interfaces is confirmed by the experiments, as can be seen in Figure 15 in the side-view photos taken from two coupons during the interruption of the tests. In the photos, the one-sided extensive delamination at the neighboring 45°/90° and 90°/45° interfaces is clearly visible. Also, at the later stages of fatigue loading, matrix cracking is also visible, as, for instance, in Figure 15a at 100,000 cycles.

### 4.4. Residual Tensile Strength

**Figure 16:**
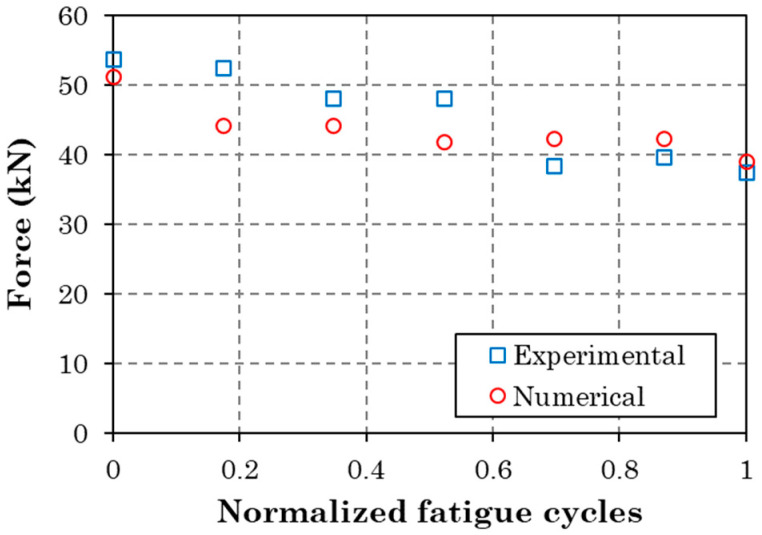
Experimental vs. numerical residual tensile strength values.

Combining the fatigue delamination model and the PDM, the residual tensile strength of the coupons was predicted. The numerical predictions are compared with the experimental measurements in Figure 16. As expected, both methods show a degradation of residual tensile strength with increasing fatigue cycles. The numerical values deviate from the experimental ones for the percentages of fatigue life of 20%, 40%, and 60%. From 0.6 to 1.0, a very good agreement is observed between the two methods. The main difference between the model and the tests is that the model considers delamination, thus neglecting all other fatigue damage modes such as matrix cracking and fiber/matrix interface failure. However, this assumption should have led to an overestimation of residual tensile strength, which is not the case. The fact that the model underestimates the residual tensile strength for the percentages of fatigue life of 20%, 40%, and 60% reveals that the neglected failure modes have not accumulated significantly at those stages and that the modeling approach for delamination (nodes release) is too severe. Moreover, the good agreement at high fatigue stages is because the delamination in the tested coupons has become severe.

**Figure 17:**
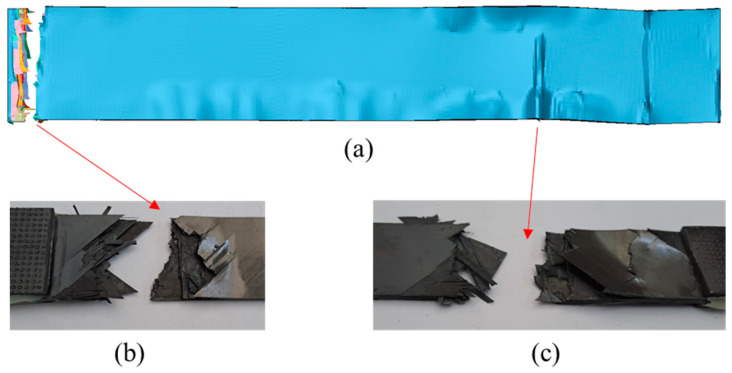
(**a**) Predicted deformed shape and fracture areas of a coupon subjected to tension after it has been fatigued for 80,000 cycles, (**b**) Left fracture area in the coupon after the test, (**c**) Right surface area in the coupon after the test.

Regarding the predicted damage propagation in the coupons subjected to tension after fatigue, the pre-existing delamination is causing an extensive matrix cracking, which should have been already present if it had been considered by the fatigue model, and a fast propagation of delamination, especially close to the loaded ends. This has led to the fracturing of the coupons in two different areas located close to the two ends, as shown in Figure 17a. This finding is verified by the tested coupons (Figure 17b,c).

## 5. Conclusions

In the present work, we have proposed a numerical modeling approach for simulating fatigue delamination growth and predicting the residual tensile strength of quasi-isotropic thermoplastic coupons. The development and implementation of the models was supported by quasi-static tension tests and fatigue tests. The model is capable of simulating fatigue delamination growth at all interfaces, thus giving a detailed insight of delamination, which is a crucial failure mechanism in the fatigue of CFRPs. The respective results have been validated successfully against C-scan images taken on fatigued coupons at interruptions of fatigue tests. The fatigue model does not account for the other types of damage except for delamination. The combination of the fatigue model with the quasi-static PDM has led to accurate predictions of the residual tensile strength of the coupons. This finding is an indication that the consideration of delamination alone is sufficient for capturing the degradation of residual strength in thermoplastic coupons. The proposed modeling approach, if upscaled to be applicable to structural parts, can prove useful in damage-tolerant design and the development of structural health monitoring systems of composite structures. Additionally, a comprehensive understanding of the failure mechanisms and gradual propagation of fatigue damage in thermoplastic composite laminates could help in the use of these types of materials by the aerospace industry.

## Figures and Tables

**Table 1:** Damage modes of the Hashin and Matzenmiller progressive damage failure modes [27].

Damage Types		Fiber Damage Modes			Matrix Damage Modes		
Damage Modes		Mode 1 *j* = 1	Mode 2 *j* = 2	Mode 3 *j* = 3	Mode 4 *j* = 4	Mode 5 *j* = 5	Mode 6 *j* = 6
Moduli	$q_{ij}^{U}$	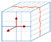	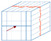	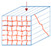	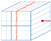	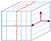	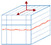
*E*_11_	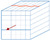	1	1	1	0	0	0
*E*_22_	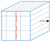	0	0	1	1	1	0
*E*_33_	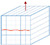	0	0	1	0	0	1
*G*_12_	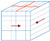	1	1	1	1	1	0
*G*_23_	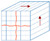	0	0	1	1	1	1
*G*_13_	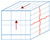	1	1	1	0	0	1

**Table 2:** The mechanical properties of the thermoplastic TC 1225 LM PAEK prepreg ply [18,21].

Mechanical Property		Value
*ρ* [ton/mm^3^]	Density	1.75 × 10^−9^
*E*_11_ [MPa]	Youngs’ Modulus—longitudinal direction	95,000
*E*_22_ [MPa]	Youngs’ Modulus—transverse direction	8500
*E*_33_ [MPa]	Youngs’ Modulus—through thickness direction	8500
*v*_21_ [-]	Poison’s ratio in 21 direction	0.027
*v*_31_ [-]	Poison’s ratio in 31 direction	0.024
*v*_32_ [-]	Poison’s ratio in 32 direction	0.172
*G*_12_ [MPa]	Shear Modulus in 12 direction	4300
*G*_23_ [MPa]	Shear Modulus in 23 direction	3571.4
*G*_13_ [MPa]	Shear Modulus in 13 direction	4300
*S_1T_* [MPa]	Longitudinal tensile strength	4000
*S_2T_* [MPa]	Transverse tensile strength	150
*S_3T_* [MPa]	Trough thickness tensile strength	300
Traction–Normal direction [MPa]	-	86
Traction–Transverse direction [MPa]	-	42
*G_I,c_* [N•mm]	Strain energy release rate of Mode I	2.1
*G_II,c_* [N•mm]	Strain energy release rate of Mode II	2.6

## Data Availability

Restrictions apply to the datasets.
